# Tannins, Peptic Ulcers and Related Mechanisms

**DOI:** 10.3390/ijms13033203

**Published:** 2012-03-08

**Authors:** Neyres Zinia Taveira de Jesus, Heloina de Souza Falcão, Isis Fernandes Gomes, Thiago Jose de Almeida Leite, Gedson Rodrigues de Morais Lima, Jose Maria Barbosa-Filho, Josean Fechine Tavares, Marcelo Sobral da Silva, Petrônio Filgueiras de Athayde-Filho, Leonia Maria Batista

**Affiliations:** Department of Pharmaceutical Sciences, Federal University of Paraiba, João Pessoa 58051-970, PB, Brazil; E-Mails: neyresj@hotmail.com (N.Z.T.J.); heloinafalcao@yahoo.com.br (H.S.F.); isisfarmacia@hotmail.com (I.F.G.); thiago454@yahoo.com.br (T.J.A.L.); gedson@ltf.ufpb.br (G.R.M.L.); jbarbosa@ltf.ufpb.br (J.M.B.-F.); josean@ltf.ufpb.br (J.F.T.); marcelosobral@ltf.ufpb.br (M.S.S.); athayde-filho@ltf.ufpb.br (P.F.A.-F.)

**Keywords:** tannins, antiulcer activity, gastric ulcer, natural products, *Helicobacter pylori*

## Abstract

This review of the current literature aims to study correlations between the chemical structure and gastric anti-ulcer activity of tannins. Tannins are used in medicine primarily because of their astringent properties. These properties are due to the fact that tannins react with the tissue proteins with which they come into contact. In gastric ulcers, this tannin-protein complex layer protects the stomach by promoting greater resistance to chemical and mechanical injury or irritation. Moreover, in several experimental models of gastric ulcer, tannins have been shown to present antioxidant activity, promote tissue repair, exhibit anti *Helicobacter pylori* effects, and they are involved in gastrointestinal tract anti-inflammatory processes. The presence of tannins explains the anti-ulcer effects of many natural products.

## 1. Introduction

Tannins are poly-phenols present in plants, foods and beverages, and are of great economic and ecological interest [1–7]. They are water soluble and with molecular weights ranging between 500 and 3000 Daltons. They also form complexes with water-insoluble proteins, alkaloids and gelatin. They are responsible for the astringent taste of many fruits and vegetables, causing precipitation of salivary glycol-proteins and reducing oral lubrication [8].

Being phenolic compounds, tannins are chemically reactive and form inter and intra-molecular hydrogen bonds. They are easily oxidized by specific plant enzymes and influenced by metals such as ferric chloride, (which causes a darkening solution). Classically, the chemical structures of tannins are divided into two groups: hydrolysable, and condensed. The hydrolysable tannins consist of gallic acid esters, and ellagic acid glycosides, formed from shikimate, where the hydroxyl sugar groups are esterified with phenolic acids [9].

Ellagitannins are much more frequent in nature than gallic tannins, and it is likely that the hexahydroxydiphenílic biphenyl acid system results from oxidative coupling between two gallic acids. Largely found in the plant kingdom, condensed tannins or proanthocyanidins are polymers of flavan-3-ol and/or flavan-3,4-diol products of phenylpropanol metabolism [10]. Proanthocyanidins, (probably so named because of red pigments from the classes of anthocyanidins, cyanidin and delphinidin), have a rich structural diversity resulting from substitutions between flavan units, a great diversity of positions, connections, and compound stereochemistry [9].

Many plant species producing tannins are used in folk medicine for different purposes. The tannin’s drug applications are mainly related to their astringent properties. They exert internal anti-diarrheal and antiseptic effects by waterproofing the outer layers of more exposed mucous membranes. Precipitating proteins, tannins provide antimicrobial and antifungal effects. Tannins are also haemostatic, and can serve as an antidote in poisoning cases [8]. In the process of healing wounds, burns and inflammations, tannins help by forming a protective layer (tannin-protein/tannin-polysaccharide complex), over injured epithelial tissues permitting the healing process below to occur naturally [9]. Studies show that many tannins act as radical scavengers, intercepting active free radicals [9], various degenerative diseases such cancer, multiple sclerosis, atherosclerosis and aging process itself are associated with high concentrations of intercellular free radicals.

In the course of our continuing search for naturally bioactive products from plants, we have published plant extract and compound reviews demonstrating various activities such as: inhibition of mammary, cervical uterine, and ovarian neoplasia [11–13]; inhibition of hydroxy-3-methyl-glutaryl (HMG) CoA reductase, of angiotensin-converting enzyme (ACE), and of acetylcholinesterase (AChE) [14–16]; of convulsion, and anxiety disorders [17,18]; central analgesic activity [19] a treatment for Parkinson’s disease [20]; a preventative for osteoporosis [21]; an antileishmanial [22]; a giardicide [23]; an anti-leprotic [24]; an anti-hypoglycemic [25] an anti-inflammatory [26–29]; a malaria treatment [30]; anti-ulcer activities [31] and effects on HIV-1 Protease [32]. Our group has also reviewed both poisonous and medicinal plants in Northeastern Brazil [33,34], among others [35–54].

In a previous paper, this research group reviewed alkaloids and flavonoids with anti-ulcer activity [55,56]. The aim of this study is to review the literature on the bioactivity of tannins against the peptic ulcer.

## 2. Pathophysiology of the Peptic Ulcer

Peptic ulcer is one of the world’s major gastro-intestinal disorders, embracing both gastric and duodenal ulcers, and affecting 10% of the world population [57]. The patho-physiology of peptic disease is attributed to the imbalance between aggressive factors like acid, pepsin, and Helicobacter infection, and the local mucosa defenses like bicarbonate secretion, mucus and prostaglandins [58]. *Helicobacter pylori* infection, use of non-steroidal anti-inflammatory drugs-NSAIDs, emotional stress, alcohol abuse, and smoking are the principal etiological factors associated with peptic ulcer [59].

In *Helicobacter pylori* infections a gram negative bacterium colonizes the human stomach, and is a risk factor for the development of peptic ulcer and gastric adenocarcinoma [60]. The vacuolating cytototoxin (VacA) is a major virulence factor, and causes cell vacuolation and subsequent tissue damage [61,62]. Other bacterial factors also involved in the development of peptic ulcers are cytotoxin-associated gene island pathogenicity (CagA), lipopolysaccharides, flagellin and urease [62].

Tissue damage to the gastrointestinal mucosa (or hemorrhagic injury) is produced by exogenous compounds as well, mainly NSAIDs and ethanol [63]. NSAIDs damage the stomach by suppressing synthesis of gastric prostaglandins. Gastric acid exacerbates NSAID effects by deepening superficial lesions, interfering with platelet aggregation, and impairing the ulcer healing process [59].

The suppression of stomach acid secretions is a key therapeutic target for ulcers, and includes the use of antacids, specific muscarinic M1 receptor antagonists, targeting gastrin receptors and histamine H2 receptors, and the use of proton pump inhibitors [58].

The exposure of gastric mucosa to aggressive factors such as absolute ethanol, stress, and ischemia followed by reperfusion, and the use of NSAIDs produce pathological changes and the development of inflammation, hemorrhagic erosions, and ulcers with the acute involvement of free radicals, or Reactive Oxygen Species (ROS) [64–66]. These radicals are normally neutralized by the action of the antioxidant system consisting of organic substances containing thiol groups such as glutathione, vitamins C and E, NADPH, antioxidant enzymes such as peroxidase, superoxide dismutase, glutathione peroxidase, glutathione reductase and others [67]. When there is an imbalance between ROS and the antioxidant defense mechanisms, ROS lead to oxidative modifications in the cellular membrane and intracellular molecules resulting in peroxidation of membrane lipids, accumulation of lipid peroxides, and cellular damage [68].

Mucosal defensives are nitric oxide-NO [69], mucus [70], bicarbonate [71] gastrin [72] and prostaglandins [73], as well mucosal blood flow [74].

## 3. Plants with Peptic Anti-Ulcer Activity

Plants rich in tannins have been traditionally used for their medicinal effects and several studies have demonstrated their anti-ulcer effects.

Annuk *et al*. (1999) investigated the effect of the leaves (aqueous extract) of *Arctostaphylos uva-ursi* (Ericaceae) and *Vaccinium vitis-idaea* (Ericaceae), for susceptibility of ten strains of *Helicobacter pylori*. It was established that the extracts were clearly bacteriostatic [75].

Perera *et al*. (2001) compared the effect of aqueous bark extract from *Rhizophora mangle* (Rizophoraceae) against cimetidine on gastric ulceration induced by ethanol- hydrochloric acid in rats, determining the quality and quantity of the mucus. The extract inhibited ulceration and promoted higher mucus volumes [76]. Berenguer *et al*. (2006) determined its effects in a model of diclofenac-induced ulcer in rats. Pretreatment with *Rhizophora mangle* resulted in a significant decrease of the ulcerated area, with increases in glutathione peroxidase and superoxide dismutase activity [77]. The authors suggest that the gastro protective effect of the extract in this experimental model is antioxidant and prostaglandin dependent.

Gonzales *et al*. (2001) conducted studies with aqueous methanolic extract from the leaves of *Maytenus aquifolium* (Celastraceae), *Soroceae bomplandii* (Moraceae), and *Zolernia ilicifolia* (Fabaceae) evaluating anti-ulcer activity through ethanol and indomethacin/bethanecol ulcer induction in mice. *Maytenus aquifolium* lowered all ulcerogenic parameters in the ethanol test. *Soroceae bomplandii* produced anti-ulcerogenic effects in both experimental models, while *Zolernia ilicifolia* showed significant effects only for indometacin/bethanecol-induced gastric lesions [78].

Martins *et al*. (2002) evaluated the anti-ulcer activity of acetone soluble fraction (AFSAB) from bark extract of *Styphnodendron adstringens* (Leguminosae), in acute models of gastric ulceration, and for basal and bethanecol-stimulated gastric acid secretion in rats. AFSAB promoted significant decreases in gastric lesions from ethanol and hypothermic restraint-stress, and significantly decreased the basal as well as bethanecol-stimulated gastric secretory volume, and total acidity [79].

Rafhael and Kuttan (2003), demonstrated elevated levels of glutathione (GSH) gastric mucosa, and ethanol lesion inhibition in rats using methanolic extract from *Phyllanthus amarus* (Euphorbiacea) [80]. GSH is a well-known antioxidant abundantly present as a low-molecular mass thiol in most organisms [81].

Khennouf *et al*. (2003) examined the gastro-protective effects of 70% acetone leave extracts of *Quercus suber* and *Quercus coccifera* (Fagaceae), as well as tannins purified from these extracts, in mice and rabbits using an ethanol-induced gastric ulcer model. Both extracts, as well as the purified tannins prevented the formation of stomach lesions and strongly inhibited lipid peroxidation in rabbit brain homogenate. The authors suggest that the gastro-protective effects are related to the anti-lipoperoxidant properties [82].

Hiruma-Lima *et al*. (2006) investigated hydroalcoholic extract (HE) of *Qualea grandiflora* (Vochysiaceae) bark in both acute and sub-acute gastric ulcer models in rodents. The oral administration of HE exhibited anti-ulcer activity in HCl/ethanol, indomethacin/bethanecol, and stress models [83]. Shay (1945) [84], showed that HE alone reduced the severity of gastric lesions. When given by intra-duodenal route, HE changes the pH, but does not modify others parameters of the gastric juice. HE presented healing activity in sub-acute gastric ulcers [83].

Andreo *et al.* (2006) evaluated methanolic (MeOH) and dichloromethane (DCM) extracts from the leaves of *Mouriri pusa* (Melastomataceae) in gastric ulcer, (HCl/ethanol, absolute ethanol, non-steroidal anti-inflammatory drug, stress, and pylorus ligature) models in mice and rats. The best results were obtained after pretreatment with MeOH extract, DCM extract did not show significant anti-ulcerogenic activity. The mechanism involving the anti-ulcerogenic activity of MeOH extract seems to be related to NO generation, with participation by an endogenous sulfhydryl group [85]. Vasconcelos *et al*. (2008) showed positive data in both 14 and 30 days of treatment with this extract [86] and Vasconcelos *et al*. (2010) investigated the effect of a tannins fraction from *Mouriri pusa* leaves (methanolic extract) on the prevention, and healing of gastric ulcers. The tannins fraction reduced the lesion area while promoting a larger regenerative mucosa which implies both gastro protective effects and healing enhancement [87].

Zayachkivska *et al*. (2006) investigated the gastro-duodenal protective effects of proanthocyanidins (PA) from *Viburnum opulus* (Caprifoliaceae) on stress-induced gastrointestinal damage. The study showed that the PA exert potent protective activity, via increases in NO generation, suppression of lipid peroxidation, augmented antioxidant activity, and changes in the glycol-conjugate content of gastro-duodenal mucosa in rats [88].

Pre-clinical trials of *Emblica officinalis* Gaertn (Euforbiaceae), known as Indian gooseberry or amla—which is notably the most important medicinal plant in the Indian traditional system of medicine—the Ayurveda have shown that this species possesses a wide spectrum of pharmacological properties. Experiments have shown that amla possesses a gastroprotective effect in addition to antioxidant activity, anti-inflammatory and free radical scavenging. These pharmacological properties are directly linked to the chemical compounds. Several studies suggest that it contains tannins, alkaloids, and phenolic compounds [89].

## 4. Purified Tannins and Peptic Antiulcer Activity

Tannins are used in medicine primarily because of their astringent properties; they react with the proteins of the tissue layers. Tannins precipitate micro proteins at the site of the peptic ulcer, forming a protective pellicle that prevents absorption of toxic substances, and promote resistance to the action of proteolytic enzymes, an associated activity against *Helicobacter pylori* [86].

Murakami *et al*. (1991) showed that ellagic acid is a potent competitive inhibitor of gastric H^+^, K^+^-ATPase, and proposed that ellagic acid may compete with ATP at the ATP hydrolysis site, thus markedly inhibiting acid secretion, and stress-induced gastric lesions [90]. Enzyme inhibition was also evident for tannic acid [91].

Khennouf *et al*. 2003 examined the gastroprotective effects of tannins purified from *Quercus suber* and *Quercus coccifera* (pedunculagin, phillyraeoidin A, castalagin and acutissimin B) on ethanol-induced gastric lesions in mice, and concluded that the protection afforded by these substances was very high, and might be due to the inhibition of acid secretion [82].

Purified tannins were tested against *Helicobacter pylori* by Funatogawa *et al*. (2004). Twenty hydrolysable tannins, 3 catechin and 6 proanthocyanidins were tested. All of the hydrolysable tannins tested demonstrated promising antibacterial activity against *Helicobacter pylori* [92].

In several experimental models of gastric ulcer, purified tannins have shown to be involved with gastrointestinal tract anti-inflammatory actions, promotion of tissue repair, acid secretion inhibition, and to present both antioxidant and anti-*Helicobacter pylori* activity (Table 1).

## 5. Material and Methods

In the present work, the anti-ulcer activity of the plants and tannins was searched through the data bank of the University of Illinois in Chicago, the NAPRALERT (Acronym for Natural Products ALERT), and the sites ScienceDirect and Pubmed. The data were updated in April 2011, using anti-ulcer plants, or tannins in the legend. The tannins and references selected for this work were also consulted as to details for both models and mechanisms.

## 6. Conclusions

Scientific literature demonstrates that tannins are involved in the anti-ulcer activities of several medicinal plants. Purified substances from these secondary tannic metabolites exhibit activity in experimental models both *in vivo* and *in vitro* for the peptic ulcer. The presence of these phenolic compounds would explain the anti-ulcer benefits of numerous natural products.

## Figures and Tables

**Table 1:** Chemical structures and action of purified tannins in the peptic ulcer.

Tannin	Chemical structure	Model assay/way of route/dose	Organism tested	Activity	Ref.
**Acutissimmin**	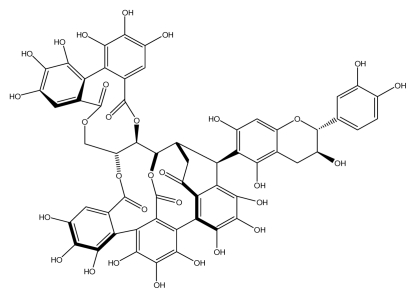	Ethanol-induced ulcers/Intragastric/50.0 mg/kg	Mouse	Active	[82]
**Agrimoniin**	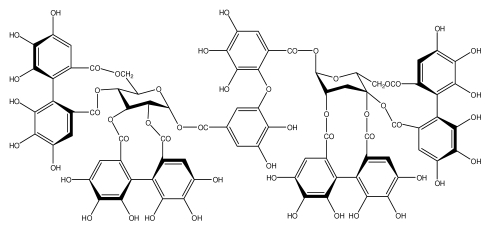	*Helicobacter pylori*-MIC (25 μg/mL)	*In vitro*	Active	[92]
**Alienanin B**	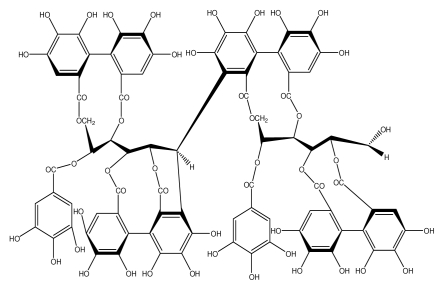	*Helicbacter pylori*-MIC (25 μg/mL)	*In vitro*	Active	[92,93]
**Chlorogenic acid**	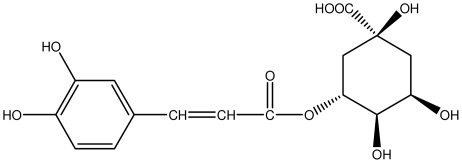	*Helicobacter pylori*-MIC (>100 μg/mL)	*In vitro*	Inactive	[92,94]

**Castalagin**	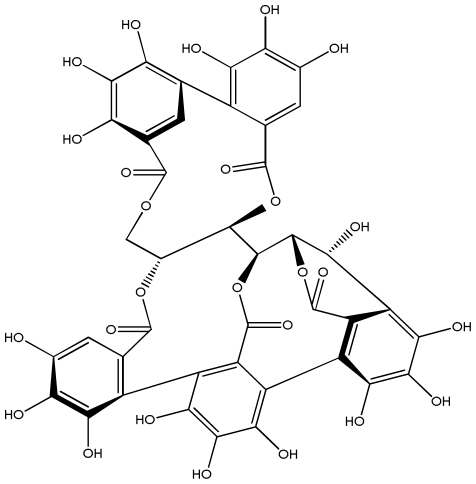	Ethanol-induced ulcers/Intragastric/50.0 mg/kg	Mouse	Active	[82,95]
**Casuarictin**	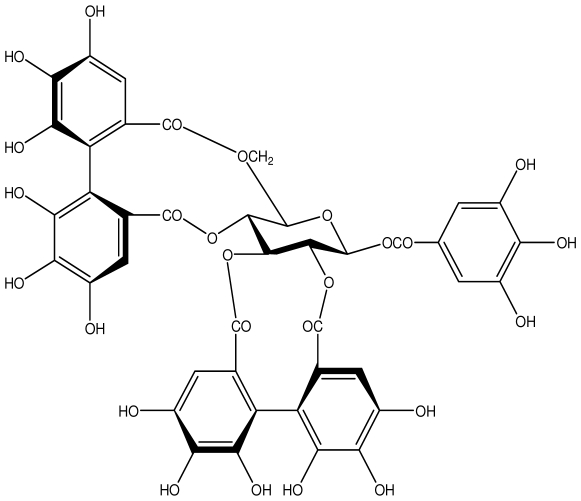	*Helicobacter pylori*-MIC (12.5 μg/mL)	*In vitro*	Active	[92,96]
**Casuarinin**	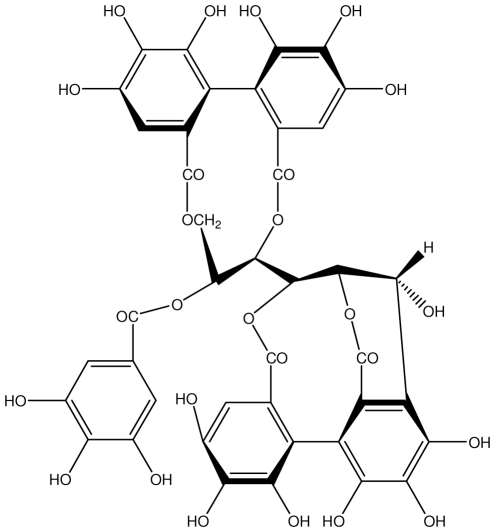	*Helicobacter pylori*-MIC (12.5 μg/mL)	*In vitro*	Active	[92,97]
**Corilagin**	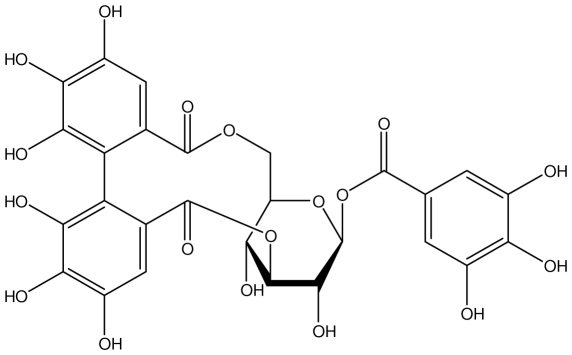	*Helicobacter pylori*-MIC (6.25 μg/mL)	*In vitro*	Active	[92,98]
**8-CRHA-Glc naringenin**	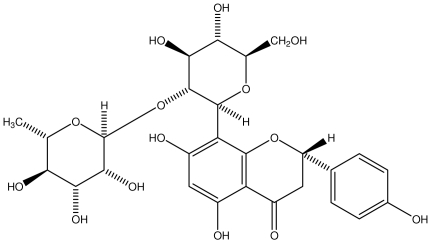	*Helicobacter pylori*-MIC (>100 μg/mL)	*In vitro*	Inactive	[92,99]
**Elaeagnatin A**	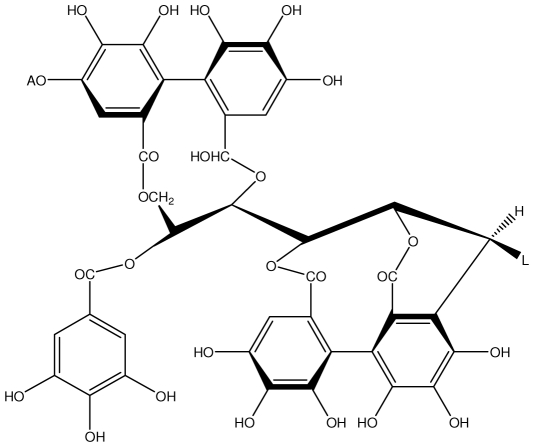	*Helicobacter pylori*-MIC (25 μg/mL)	*In vitro*	Active	[92,100]
**Elagic acid**	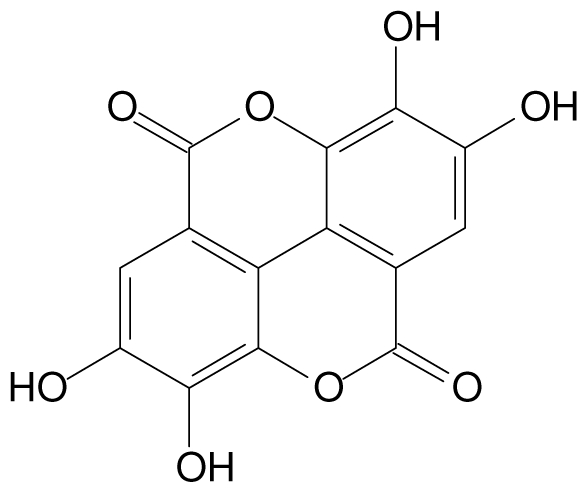	Stress-induced ulcers(water immersion)/intraperitoneal/5, 10 and 25 mg/kg	Rat	Active	[90,101]
		Pylorus-ligated animals/Intraperitoneal/5, 10 and 25 mg/kg	Rat	Active	[90,101]
		Inhibition of gastric H+, K+-ATPase	Hog gastric mucosal	Active	[90,101]
**Epicatechin**	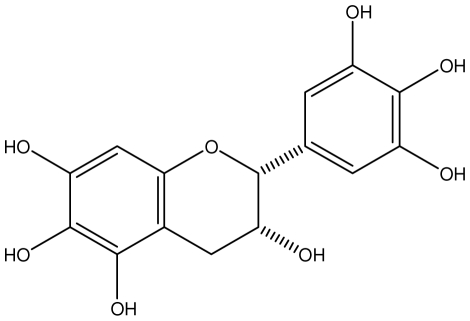	*Helicobacter pylori*-MIC (>100 μg/mL)	*In vitro*	Inactive	[92,102]
**Epicatechin gallate**	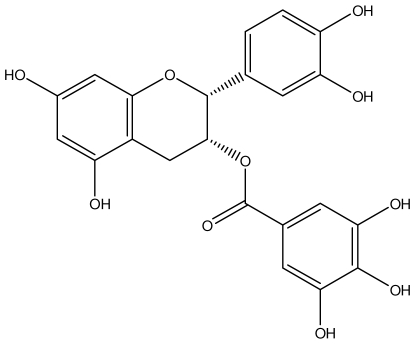	*Helicobacter pylori*-MIC (50 μg/mL)	*In vitro*	Active (Less)	[92,102]
**Epigallocatechin gallate**	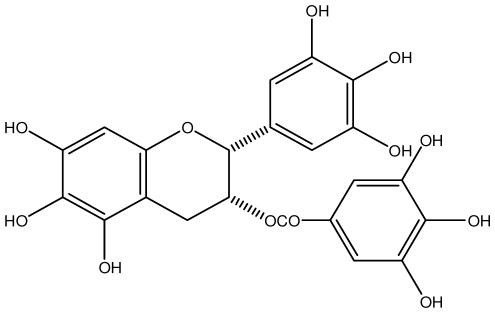	*Helicobacter pylori*-MIC (25 μg/mL)	*In vitro*	Active	[92,102]
**Geraniin**	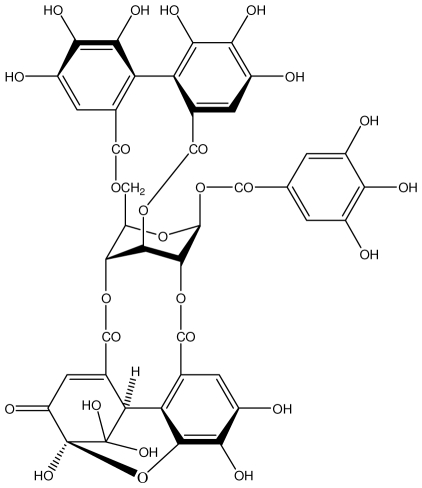	*Helicobacter pylori*-MIC (12.5 μg/mL)	*In vitro*	Active	[92]
		Stress induced ulcer	Mouse	Active	[92,103]
**Heterophylliin G**	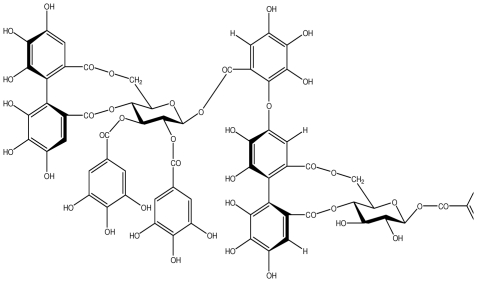	*Helicobacter pylori*-MIC (12.5 μg/mL)	*In vitro*	Active	[92,104]
**Hippophenin A**	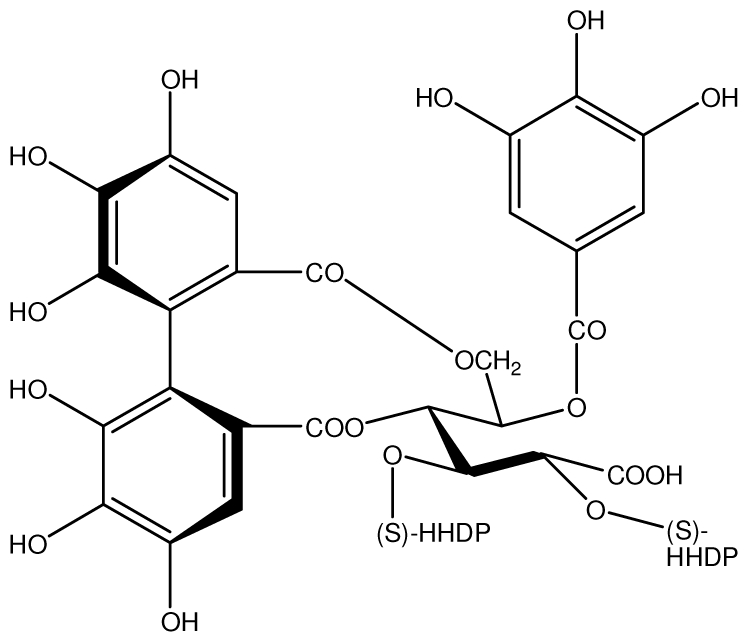	*Helicobacter pylori*-MIC (12.5 μg/mL)	*In vitro*	Active	[92,100]
**Iridin**	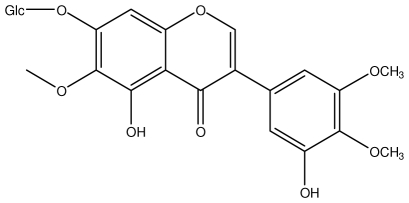	*Helicobacter pylori*-MIC (>100 μg/mL)	*In vitro*	Inactive	[92,105]
**Isorhamnetin 3-*O*-rutinoside**	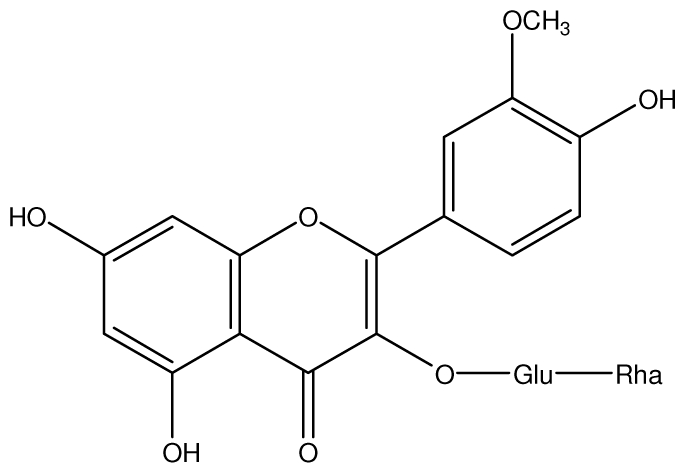	*Helicobacter pylori*-MIC (>100 μg/mL)	*In vitro*	Inactive	[92,106]
**Nobotanin B**	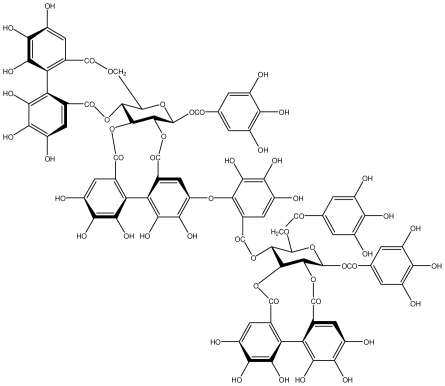	*Helicobacter pylori*-MIC (12.5 μg/mL)	*In vitro*	Active	[92,96]
**Oenothein A**	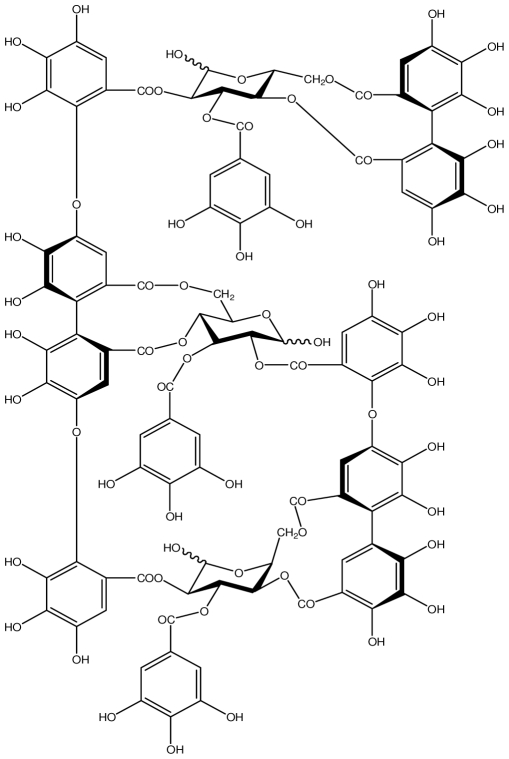	*Helicobacter pylori*-MIC (12.5 μg/mL)	*In vitro*	Active	[92]
**Oenothein B**	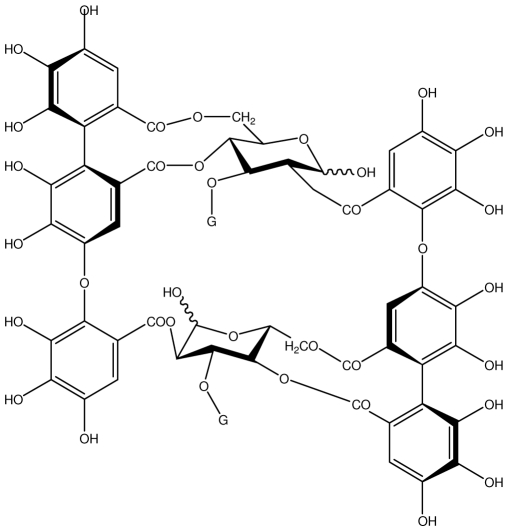	*Helicobacter pylori*-MIC (12.5 μg/mL)	*In vitro*	Active	[92,107]
**Pedunculagin**	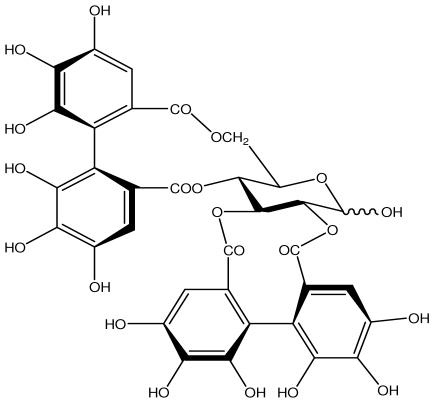	Ethanol-induced ulcers/Intragastric/50.0 mg/kg	Mouse	Active	[82,108]
**Penta-*O*-galloyl-β-d-glucose**	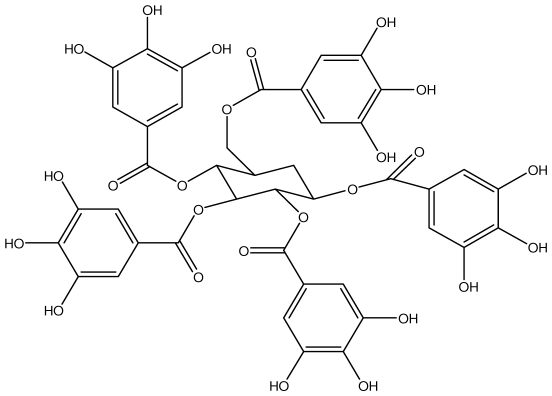	*Helicobacter pylori*-MIC (12.5 μg/mL)	*In vitro*	Active	[92,109]
**Phillyraeoidin A**	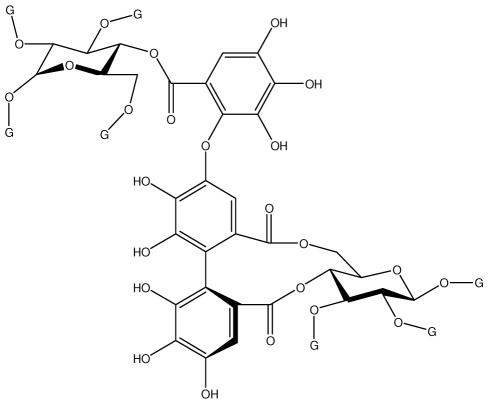	Ethanol-induced ulcers/Intragastric/50.0 mg/kg	Mouse	Active	[82,110]

**Procyanidin B1**	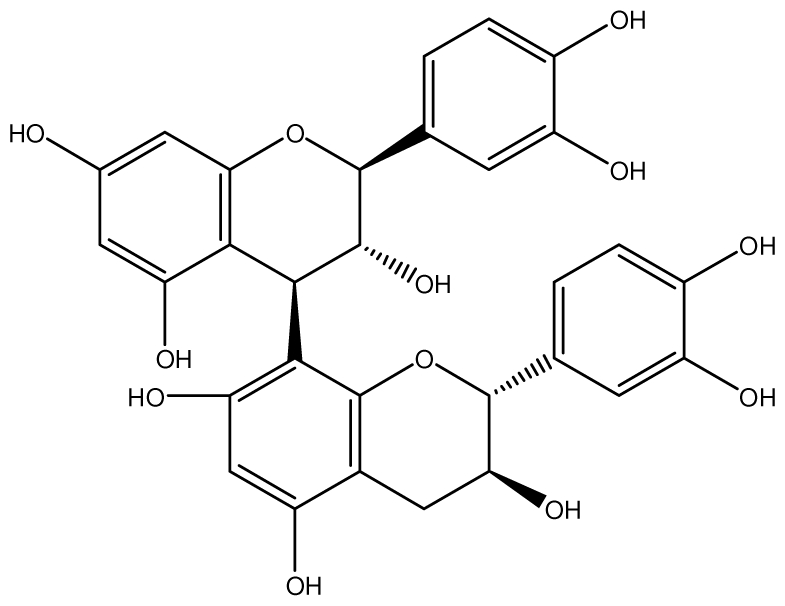	*Helicobacter pylori*-MIC (>100 μg/mL)	*In vitro*	Inactive	[92,111]
**Procyanidin B3**	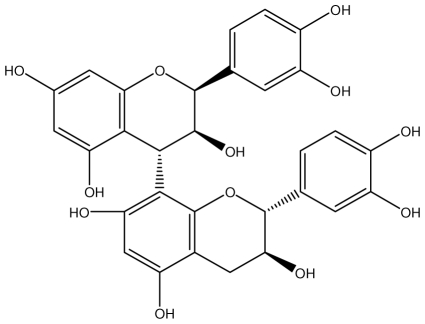	*Helicobacter pylori*-MIC (50 μg/mL)	*In vitro*	Minimal activity	[92,111]
**Procyanidin B4**	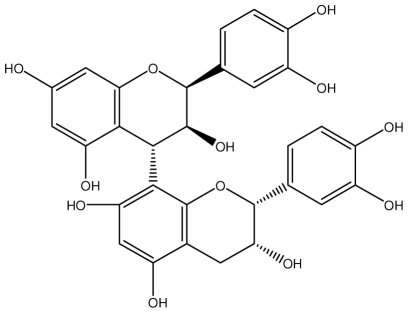	*Helicobacter pylori*-MIC (50 μg/mL)	*In vitro*	Minimal activity	[92,111]
**Procyanidin B5**	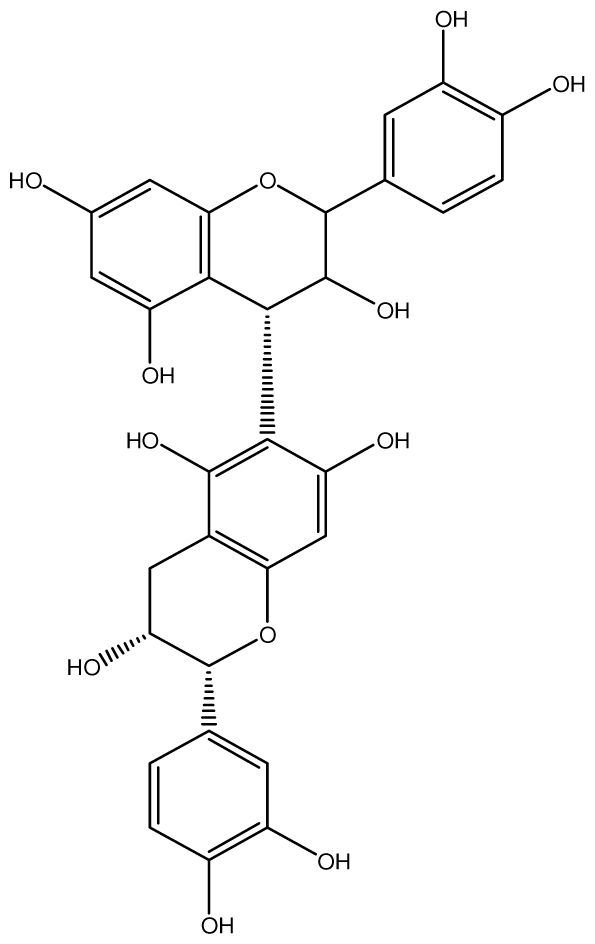	*Helicobacter pylori*-MIC (25 μg/mL)	*In vitro*	active	[92,112]
**Procyanidin C1**	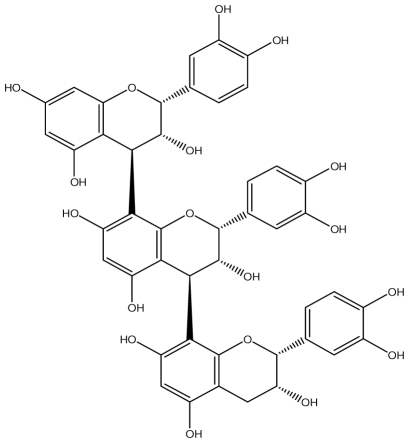	*Helicobacter pylori*-MIC (>100 μg/mL)	*In vitro*	Inactive	[92,113]
**Procyanidin polymer**	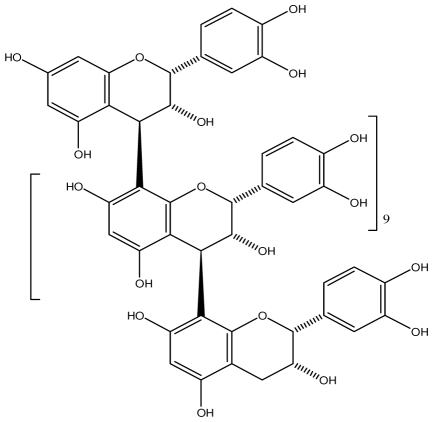	*Helicobacter pylori*-MIC (>100 μg/mL)	*In vitro*	Inactive	[92,99]
**Rugosin D**	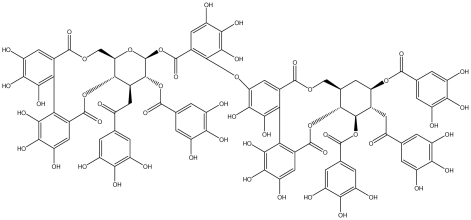	*Helicobacter pylori*-MIC (25 μg/mL)	*In vitro*	Active	[92]
**Strictinin**	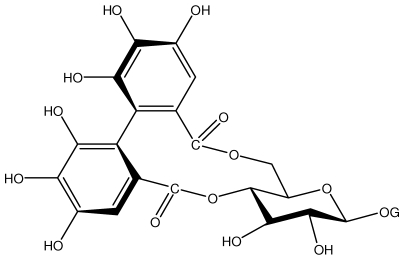	*Helicobacter pylori*-MIC (6.25 μg/mL)	*In vitro*	Active	[114]
**Tannic acid**	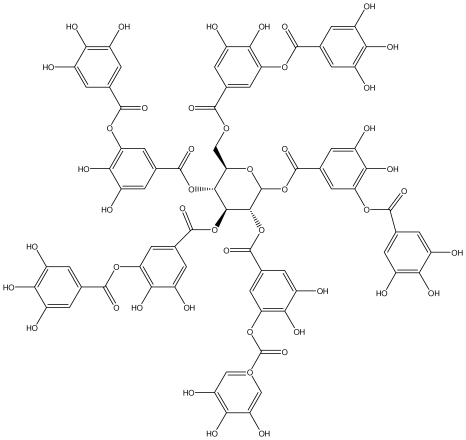	Shay ulcer/oral/50.0 mg/kg	Rat	Active	[115,116]
		Acetic acid-induced ulcer/oral/200.0 mg/kg	Rat	Active	[116]
		Pylorus-ligated animals/oral/50.0, 100.0 and 500 mg/kg	Rat	Active	[116]
		Ethanol induced gastric lesions/gastric intubation/100.0 mg/kg	Rat	Active	[117]
		Inhibition of gastric H^+^, K^+^-ATPase	Hog gastric mucosal	Active	[118]
**Tellimagrandin I**	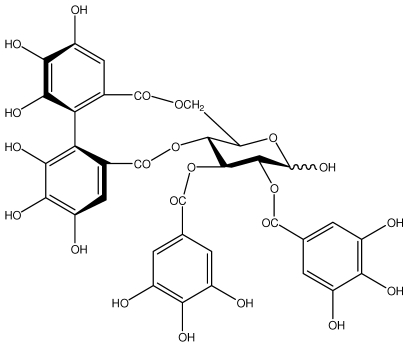	*Helicobacter pylori*-MIC (12.5 μg/mL)	*In vitro*	Active	[92]

**Tellimagrandin II**	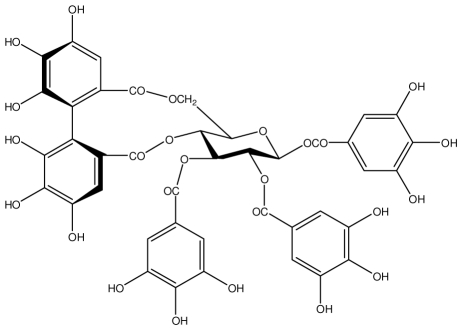	*Helicobacter pylori*-MIC (6.25 μg/mL)	*In vitro*	Active	[92]
**Tri-*N-*coumaroyl-spermidine**	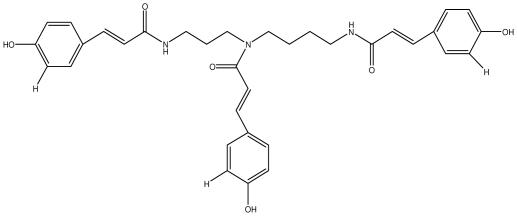	*Helicobacter pylori*-MIC (>100 μg/mL)	*In vitro*	Inactive	[92,118]
